# Managing Drawbacks in Unconventional Successful Glaucoma Surgery: A Case Report of Stent Exposure

**DOI:** 10.1155/2015/847439

**Published:** 2015-07-29

**Authors:** Antonio Fea, Paola Maria Loredana Cannizzo, Giulia Consolandi, Carlo Alessandro Lavia, Giulia Pignata, Federico M. Grignolo

**Affiliations:** Dipartimento di Scienze Chirurgiche, Clinica Oculistica dell'Università, Via Juvarra 19, 10122 Torino, Italy

## Abstract

Traditional options in managing failed trabeculectomy (bleb needling, revision, additional incisional surgery and tube surgery) have a relatively high failure and complication rate. The use of microinvasive glaucoma surgery (MIGS) has generally been reserved to mild to moderate glaucoma cases, proving good safety profiles but significant limitations in terms of efficacy. We describe a patient who underwent MIGS (XEN Aquesys subconjunctival shunt implantation) after a prior failed trabeculectomy. After the surgery, the IOP was well controlled but as the stent was close to an area of scarred conjunctiva of the previous trabeculectomy, it became partially exposed. As a complete success was achieved, we decided to remove the conjunctiva over the exposed area and replace it by an amniotic membrane transplantation and a conjunctiva autograft. Six months after surgery, the unmedicated IOP is still well controlled with complete visual acuity recovery.

## 1. Introduction

Despite its widespread use, trabeculectomy has some limitations. Aside from short-term problems such as a near 50% rate of transient perioperative complications [1], the long-term rate of failure has been reported to be as high as 50% at 5 years, even with adjunctive 5-FU or MMC, as reported in “the tube versus trabeculectomy study” [2].

The main causes of a failed trabeculectomy are episcleral or subconjunctival fibrosis [3, 4]. When a trabeculectomy procedure fails, subsequent procedures may include needling, a second trabeculectomy, or placement of a tube shunt. All of these procedures have potential complications and are generally reported to have a higher failure rate than primary trabeculectomy [5].

The use of microinvasive glaucoma surgery (MIGS) has generally been confined to mild to moderate glaucoma cases, often in combination with cataract surgery, and not in more severe glaucoma patients (especially if they have undergone previous glaucoma surgery) [6].

While other MIGS devices target Schlemm's Canal and the suprachoroidal space, the XEN subconjunctival stent is the world's first ab interno MIGS approach to subconjunctival outflow.

During surgery, the 6 mm gelatin stent is preloaded into a disposable injector with a 27-gauge needle. The stent creates a permanent patent channel through the sclera allowing aqueous humor to flow from the anterior chamber to the subconjunctival space. A sub-2 mm clear cornea main incision is used for the procedure. The injector enters the incision and travels across the eye to the superior nasal quadrant where the stent will be placed. The Gel Stent is delivered by actuating the slider on the one handed injector.

Once in place, the outflow is regulated by the physics of the internal stent lumen.

Here, we report the case of a patient with a long-term failed trabeculectomy and a badly scarred superior bleb, who was subsequently implanted with the subconjunctival XEN stent.

The stent was placed nasally to the area of the previous bleb. Despite being successful in reducing pressure, the stent became exposed due to extremely thin and weak conjunctiva and also due to the nasal location. This case briefly explains how we rescued the surgery, by patching the stent with amniotic membrane and a graft of conjunctiva. Six months after surgery, the IOP is under control without medications.

## 2. Case Report

S.A. is a 51-year-old Caucasian male who underwent a trabeculectomy in his left eye in 2004 at another center. He is a high myope, −11.5 spherical equivalent, with a visual acuity of 0,4. In October 2014, he presented a severe visual field damage, with a mean deviation (MD) of −21.65 dB and a pattern standard deviation (PSD) of 11.04 dB (Humphrey 24-2 SITA Standard, Carl Zeiss Meditec, Inc., Dublin, CA). His IOP remained under control with maximal tolerated medical therapy until October 2014, at which point his IOP rose above 26 mmHg on a number of occasions, and he was ultimately scheduled for secondary surgery approach. Due to his previous complicated trabeculectomy, the patient requested a less invasive approach and XEN surgery was proposed.

In November 2014, he underwent an uncomplicated XEN surgery. Due to the presence of a scarred conjunctiva in the superior quadrants, the stent was implanted in the nasal quadrant at 9.30 o'clock rather than the usual 11 o'clock position. This abnormal position may have ultimately contributed to the exposure because the XEN stent is less protected by the superior eyelid. Other than that, it appeared to be a successful XEN surgery.

**Figure 1:**
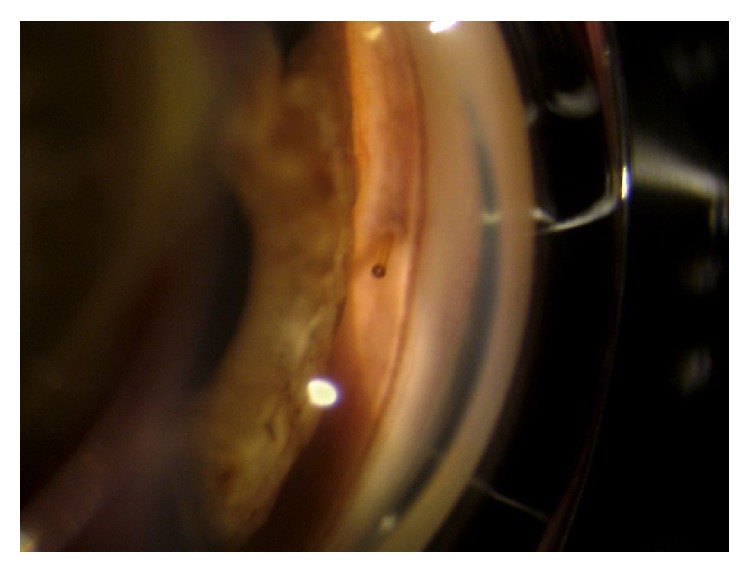
The internal bore of the stent is clearly visible into the anterior chamber.

On the first postoperative day, the bleb was well formed, the IOP was 6 mmHg, and the visual acuity was only lightly reduced to 0,3. The stent appeared well placed both subconjunctivally and at the gonioscopic examination (Figure 1).

One week postop, the IOP remained in the low teens and visual acuity fully recovered.

**Figure 2:**
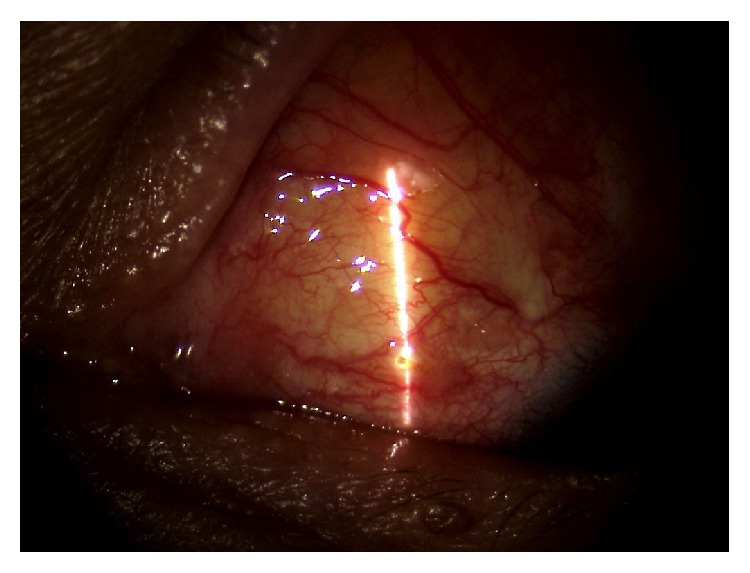
15 days after surgery, the stent was exposed in its terminal part.

At the 15 days' postoperative exam, the stent appeared exposed. The visual acuity was stable, but IOP was lower than 4 mmHg. The anterior chamber was well formed without signs of inflammation and there was no choroidal detachment (Figure 2).

We then determined to bring the patient back in for a rescue surgery. The patient underwent an amniograft with autologous conjunctival transplantation. Careful dissection of a large area of conjunctiva, from 7 to 11 o'clock, was done in the area where the stent was implanted.

**Figure 3:**
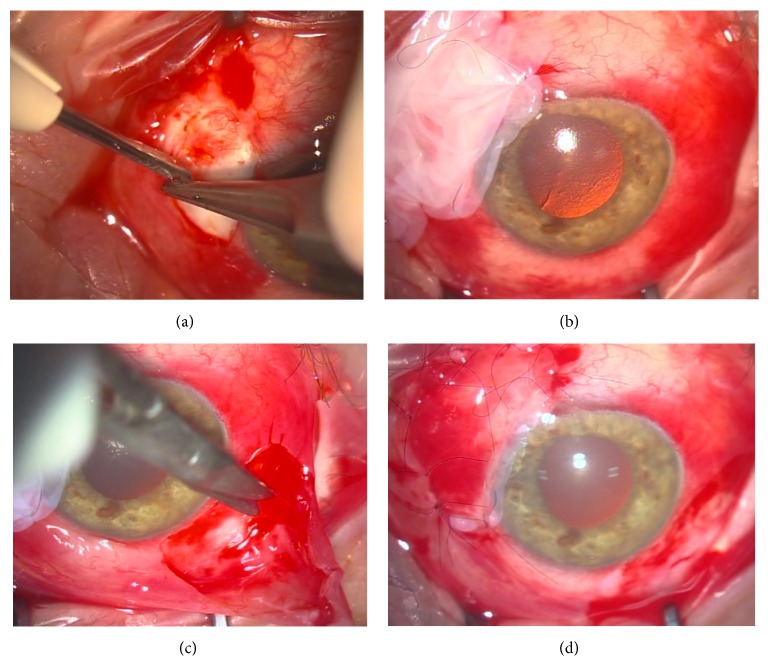
(a) A large area of conjunctiva surrounding the stent was excised and mild diathermy was applied over the scleral area. (b) A double layer of amniotic membrane was sutured over the dissected area. (c) After ballooning the conjunctiva with lidocaine, a larger patch of conjunctiva was excised in the inferotemporal quadrant to serve as a graft. (d) The graft was sutured over the amniotic membrane with 10-0 nylon interrupted sutures.

Mild diathermy was performed over the area to avoid excessive bleeding (Figure 3(a)).

The amniotic membrane folded twice keeping the epithelial side in the middle and was implanted over the area and sutured at the limbus with 10-0 nylon (Figure 3(b)).

The inferotemporal conjunctiva was then ballooned, injecting lidocaine to harvest the conjunctival graft which was sutured over the amniograft with interrupted 10-0 nylon sutures (Figures 3(c) and 3(d)).

After surgery, the patient was kept on local antibiotics and steroids.

One day later, the bleb was formed, visual acuity was 0,2, and IOP was 13 mmHg. By one week, his previous visual acuity had recovered.

The patient was followed weekly during the next month with IOP in the low teens without antiglaucoma medications.

Over the next 6 months, the patient presented with a pressure constantly lower than 14 mmHg and a stable visual acuity.

**Figure 4:**
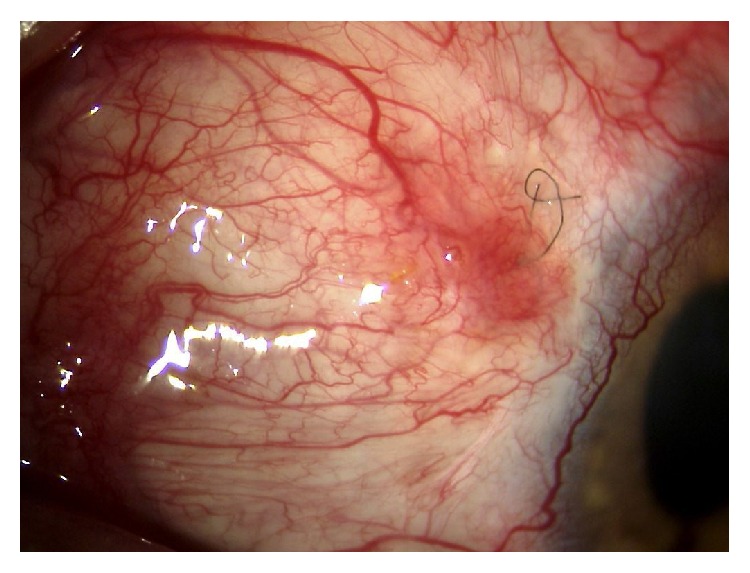
The bleb as it appears 6 months after surgery. A deep suture that could not be easily removed is still visible under the conjunctiva.

More than six months after surgery, the IOP is still under control without medications and the stent is well covered (Figure 4). At the last visual field performed, in May 2015, he presented a MD of −21.91 and a PSD of 10.98.

## 3. Discussion

Trabeculectomy is considered the standard technique for lowering intraocular pressure in glaucoma patients. In its modern version, its success rate is generally high despite being dependent on the postoperative follow-up. As time progresses after surgery, however, failure of filtration is relatively common and the management of a late failed filtering bleb is controversial.

Despite being generally advocated for early failure and needling as well as trabeculectomy revision, a second trabeculectomy or a tube implant is potential option.

With the exclusion of tube surgery, all anterior procedures, nevertheless, present a higher failure rate if compared to primary trabeculectomy.

Currently, there is no evidence based approach to a late failed bleb and the choice of surgery is influenced by the personal surgical experience as well as by the clinical appearance.

In this case report, we decided to take a new approach. Our decision was founded on previous unfortunate experience of the patient who reported to have lost several lines of visual acuity following trabeculectomy.

We proposed a less invasive approach by implanting a XEN stent and the patient agreed.

Trabecular surgery might have been another choice, but the range of pressure would have been in the midteens and our previous experience with trabecular surgery in failed trabeculectomies had generally been poor.

Although our follow-up is relatively limited, the use of XEN stent in failed trabeculectomies and our grafting techniques are both quite promising. Our case shows that XEN stent may be successful in patients with a late failed and scarred bleb. It also points out that if the implant is placed too close to the scarred tissue, too nasal, or too short, it may become exposed.

A recent Cochrane report [7] has examined the different strategies to seal a bleb leak. Out of 1333 reports, only a single study was selected for analysis, indicating that there is no clear evidence on the superiority of a single intervention. This study randomly compared amniotic membrane transplantation to conjunctival advancement and indicated a clear advantage of the latter. In our patient, conjunctival advancement would have been difficult due to the presence of scarred conjunctiva and we selected a mixed approach by transplanting a large and double patch of amniotic membrane to be a scaffold for a free conjunctival graft. The amnion presents anti-inflammatory properties and growth factors that prevent or decrease fibrosis in healing tissue [8].

The XEN implant was successful in lowering the IOP in the low teens and its minimal impact on the globe anatomy still allows other surgical options.

Because traditional glaucoma surgeries can have a limited life span and have potentially serious complications, we believe that minimally invasive surgeries may play a role in widening the sight spectrum of glaucoma patients.

## References

[1] Jampel H. D., Musch D. C., Gillespie B. W., Lichter P. R., Wright M. M., Guire K. E. (2005). Perioperative complications of trabeculectomy in the Collaborative Initial Glaucoma Treatment Study (CIGTS). *American Journal of Ophthalmology*.

[2] Gedde S. J., Schiffman J. C., Feuer W. J., Herndon L. W., Brandt J. D., Budenz D. L. (2012). Treatment outcomes in the Tube Versus Trabeculectomy (TVT) study after five years of follow-up. *American Journal of Ophthalmology*.

[3] Skuta G. L., Parrish R. K. (1987). Wound healing in glaucoma filtering surgery. *Survey of Ophthalmology*.

[4] Edmunds B., Thompson J. R., Salmon J. F., Wormald R. P. (2002). The National Survey of Trabeculectomy. III. Early and late complications. *Eye*.

[5] Saheb H., Gedde S. J., Schiffman J. C., Feuer W. J. (2014). Outcomes of glaucoma reoperations in the Tube Versus Trabeculectomy (TVT) study. *American Journal of Ophthalmology*.

[6] Saheb H., Ahmed I. I. K. (2012). Micro-invasive glaucoma surgery: current perspectives and future directions. *Current Opinion in Ophthalmology*.

[7] Bochmann F., Azuara-Blanco A. (2012). Interventions for late trabeculectomy bleb leak. *Cochrane Database of Systematic Reviews*.

[8] Lo K., Kohanim S., Trief D., Chodosh J. (2013). Role of amniotic membrane transplantation in acute chemical injury. *International Ophthalmology Clinics*.

